# RNase L Induces Expression of A Novel Serine/Threonine Protein Kinase, DRAK1, to Promote Apoptosis

**DOI:** 10.3390/ijms20143535

**Published:** 2019-07-19

**Authors:** Praveen Manivannan, Vidita Reddy, Sushovita Mukherjee, Kirsten Neytania Clark, Krishnamurthy Malathi

**Affiliations:** Department of Biological Sciences, University of Toledo, 2801 West Bancroft Street, Toledo, OH 43606, USA

**Keywords:** RNase L, DRAK1, apoptosis, interferon signaling, JNK

## Abstract

Apoptosis of virus-infected cells is an effective antiviral mechanism in addition to interferon induction to establish antiviral state to restrict virus spread. The interferon-inducible 2′–5′ oligoadenylate synthetase/RNase L pathway results in activation of RNase L in response to double stranded RNA and cleaves diverse RNA substrates to amplify interferon induction and promote apoptosis. Here we show that RNase L induces expression of Death-associated protein kinase-Related Apoptosis-inducing protein Kinase 1 (DRAK1), a member of the death-associated protein kinase family and interferon-signaling pathway is required for induction. Overexpression of DRAK1 triggers apoptosis in the absence of RNase L activation by activating c-Jun N-terminal kinase (JNK), translocation of BCL2 Associated X (Bax) to the mitochondria accompanied by cytochrome C release and loss of mitochondrial membrane potential promoting cleavage of caspase 3 and Poly(ADP-Ribose) Polymerase 1 (PARP). Inhibitors of JNK and caspase 3 promote survival of DRAK1 overexpressing cells demonstrating an important role of JNK signaling pathway in DRAK1-mediated apoptosis. DRAK1 mutant proteins that lack kinase activity or nuclear localization fail to induce apoptosis highlighting the importance of cellular localization and kinase function in promoting cell death. Our studies identify DRAK1 as a mediator of RNase L-induced apoptosis.

## 1. Introduction

Apoptosis is a regulated process of cell death that plays a critical role in many physiological events, including the cellular response to virus infection. Viral infection triggers the innate immune response involving pathogen detection and signaling to produce chemokines and cytokines, including type I interferons (IFN), to limit viral replication and spread to neighboring cells [1]. Type I IFN released by infected cells binds to interferon receptor (IFNAR) and triggers signaling pathways to induce expression of interferon-stimulated genes (ISGs) which are essential for the establishment of an antiviral state [2]. In addition, virus infection promotes apoptosis as a potent antiviral mechanism to eliminate infected cells to restrict spread in the host [3,4,5]. IFNs are essential to mount antiviral response and many of the ISGs like protein kinase R (PKR), 2′–5′ oligoadenylate synthase (OAS), interferon-stimulated gene 54 (ISG-54), retinoic acid–inducible gene I (RIG-I), Melanoma differentiation-associated gene 5 (Mda5), promyelocytic leukemia (PML), Tumor Necrosis Factor (TNF)-related apoptosis inducing ligand (TRAIL) among many others are mediators of apoptosis [3,6,7,8,9,10].

The 2′–5′-oligoadenylate synthetase (OAS)-RNase L pathway is an antiviral response that is induced by IFN and dsRNA produced during viral infections. OAS1 to three isoforms are activated by dsRNA and convert cellular ATP into 2′–5′-oligoadenylate oligomers (2–5A) that bind to the ubiquitously expressed latent endoribonuclease, Ribonuclease L (RNase L) [11]. Binding of RNase L inactive monomers to 2–5A, causes conformational change and dimerization to produce an active nuclease that cleaves single-stranded viral and cellular RNAs after UpUp or UpAp residues, producing small dsRNA products with roles in transcriptional activation of antiviral and antitumor genes, amplifying IFN production, activating inflammasomes and promoting switch from autophagy to apoptosis [12,13,14,15,16]. Activation of RNase L leads to mitochondrial release of cytochrome C and caspase-dependent apoptosis involving JNK activity in a cell-type specific manner [17,18]. Unexpectedly, RNase L transcriptionally induced antiviral and antitumor genes [12]. Among the genes that were transcriptionally induced by RNase L, death-associated protein kinase-related apoptosis-inducing protein kinase 1 (DRAK1) was a unique member of the DAP-kinase family, and its role in cell death by IFN signaling was unknown [12].

DRAK1, also known as STK17A, is a member of the death-associated protein kinase (DAPK) family of proteins and shares homology with DRAK2 (STK17B). Both proteins share homology with other DAPK members in the kinase domain, however, the regulatory domains are distinct, suggesting involvement in cell death induced by different stimuli [19]. The role of DRAK1 in inducing apoptosis varies in different cell types. Overexpression in NIH3T3 cells induced morphological changes characteristic of apoptosis [19]. In testicular cancer cells, DRAK1 is induced in a p53-dependent manner by cisplatin and induces cell death by regulating reactive oxygen species [20]. Interestingly, DRAK1 was overexpressed in glioblastoma and promoted cell proliferation, migration and resistance to genotoxic agents [21,22]. DRAK1 is overexpressed and localized in the cytoplasm in head and neck squamous cell carcinomas (HNSCC) and negatively regulates the tumor suppressor function of TGF-β by binding to Smad3 which is required for Transforming Growth Factor Beta (TGF-β) function [23]. Recently, DRAK1 was shown to localize in the nucleus and translocate to the cytoplasm in response to Protein Kinase C (PKC) activation and interact with p53 and mitochondrial protein, Adenine Nucleotide Translocator 2 (ANT2), suggesting regulation at multiple levels [24].

In this study, we show that DRAK1, and not DRAK2, is induced by RNase L and IFN signaling. Ectopic expression of DRAK1 in prostate cancer cells is sufficient to induce apoptosis in the absence of RNase L activation. Our studies show that DRAK1 induces JNK activity and cell death involving Bax translocation to the mitochondria accompanied by loss of mitochondrial membrane potential and cleavage of caspase 3 and PARP. Finally, we show that both DRAK1 nuclear localization and kinase activity are required for inducing apoptosis. These studies identify DRAK1 as one of the factors that contributes to cell death in response to RNase L activation.

## 2. Results

### 2.1. Induction of DRAK1 by RNase L Requires Interferon Signaling

**Figure 1:**
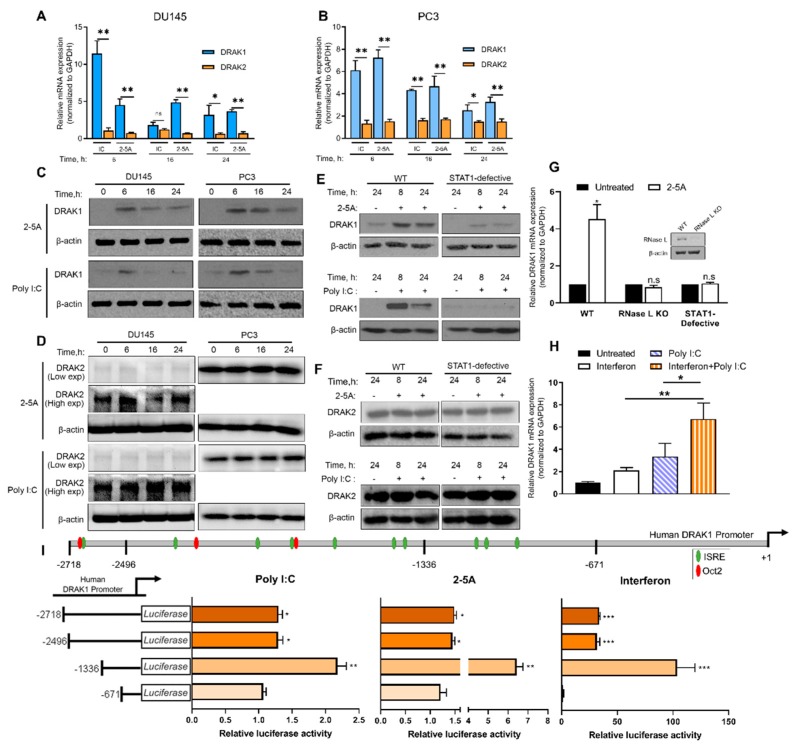
Induction of DRAK1 by RNase L activation and type I IFN signaling. DU145 and PC3 cells were transfected with Poly I:C (2 µg/mL) or 2–5A (10 µM) for indicated times and (**A**,**B**) mRNA expression of DRAK1 and DRAK2 were analyzed by qPCR, (**C**,**D**) protein expression was determined by immunoblot analysis. (**E**,**F**) DRAK1 and DRAK2 expression on immunoblots in HT1080 and U3A (STAT1-defective) cells, (**G**) mRNA levels in indicated cells were compared to untreated cells and analyzed by qPCR. Inset shows immunoblot analysis of RNase L KO cells (**H**) DRAK1 mRNA levels in PC3 levels transfected with Poly I:C (2 µg/mL), interferon (1000 U/mL) or combined treatment for 6 h, (**I**) Schematic of DRAK1 promoter region showing IFN-stimulated response element (ISRE) sequences (in green) and Octamer-Binding Protein-2 (Oct-2) binding sites (in red). DRAK1 promoter driven luciferase activity normalized to *renilla* luciferase in PC3 treated with Poly I:C (2 µg/mL) or 2–5A (10 µM) or interferon (1000 U/mL). Values shown are mean ± s.d of triplicate assays. * *p* < 0.01, ** *p* < 0.001, *** *p* < 0.0001, n.s: not significant, WT: Wild Type.

Microarray analysis of DU145 cells transfected with 2-5A to activate RNase L identified changes in mRNA levels of antiviral and antitumor genes including death-associated protein kinase (DAPK)-related apoptosis-inducing protein kinase 1 (DRAK1), a member of the death-associated protein (DAP) family of serine/threonine protein kinases. To determine if DRAK1 is induced in prostate cancer cells in response to RNase L activation, PC3 and DU145 cells were transfected with 2–5A or synthetic dsRNA, Poly I:C, which activates oligoadenylate synthetase to produce 2–5A. After 6 h, 6- or 7-fold increase in mRNA in PC3 cells and 11- or 4.5-fold in DU145 cells was observed that correlated with protein expression (Figure 1A–D). However, DRAK2, a DRAK1 homolog, was not induced by 2–5A or Poly I:C treatment. RNase L activity produces cleaved dsRNAs that amplify interferon signaling [13]. Compared to wild-type (2fTGH, fibrosarcoma) cells, DRAK1 induction was significantly reduced in STAT1-defective cells (U3A, derived from 2fTGH and defective in type I IFN signaling), while DRAK2 levels remained unchanged (Figure 1E,F). Cells lacking RNase L (inset Figure 1G) or STAT1 signaling showed reduced DRAK1 mRNA induction by 2-5A (Figure 1G). Further, treatment of cells with both Poly I:C and IFN induced DRAK1 mRNA levels 6-fold compared to 2- to 3-fold by either Poly I:C or IFN (Figure 1H). These results suggest the requirement of the IFN-signaling pathway in DRAK1 transcriptional induction by RNase L.

To determine if RNase L-induced expression of DRAK1 was due to transcriptional activation of the DRAK1 promoter, the 5′ flanking region corresponding to −2718/+1 of DRAK1 promoter was inserted upstream of a luciferase reporter gene and induction by 2–5A, Poly I:C or IFN was monitored. To define the minimum promoter region necessary for induction, promoter deletion constructs was generated. A 1336 bp promoter fragment conferred greater responsiveness to 2–5A, Poly I:C and IFN and harbored perfect consensus sequences for IFN-stimulated response element (ISRE) typically found in the promoter regions of IFN-stimulated genes (Figure 1I) [25]. The −2718 bp and −2496 bp fragments harbored ISRE sequences in addition to Oct-2 binding sites that function as transcriptional repressors [26]. Deletion of the Oct-2 binding sites in −1336 bp fragment conferred greater inducibility. However, further studies will be needed to demonstrate if Oct-2 or other negative regulatory elements regulate DRAK1 promoter activity. These findings suggest that DRAK1 is transcriptionally induced by RNase L activation and more directly by IFN.

### 2.2. DRAK1 Localizes to The Nucleus and Induces Apoptosis

**Figure 2:**
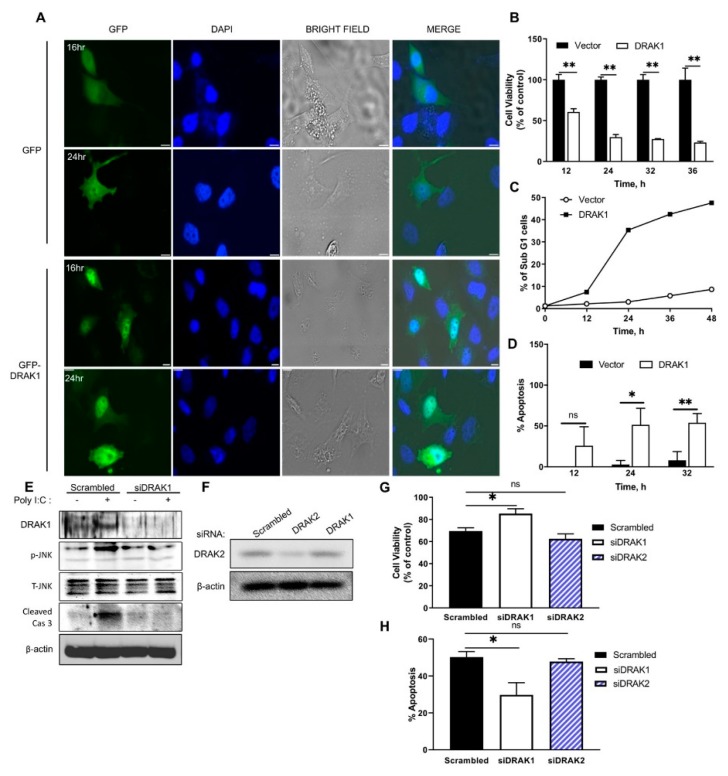
Nuclear localization of DRAK1 and induction of apoptosis (**A**) Fluorescence microscopy of DRAK-GFP or GFP vector expression and bright field images at indicated times in PC3 cells (scale bar, 10 µm, representative images are shown). PC3 cells were transfected with vector or DRAK1 construct and cell viability was determined at indicated times by (**B**) 3-(4,5-dimethylthiazol-2-yl)-2,5-diphenyltetrazolium bromide (MTT) assay and normalized to control, (**C**) uptake of Propidium Iodide (PI) by dying cells as measured by flow cytometry after staining with PI, (**D**) trypan blue dye exclusion assay normalized to control cells. Data shown as % apoptotic cells. PC3 cells were transfected with siRNA targeting DRAK1, DRAK2 or scrambled control siRNA and knock down and JNK phosphorylation and caspase 3 cleavage was determined on immunoblots following transfection with Poly I:C (**E**,**F**), and (**G**,**H**) cell viability was analyzed after transfection with 2–5A (10 µM) by MTT assay and % apoptotic cells was determined by trypan blue exclusion assay. Values shown are mean ± s.d of triplicate assays. * *p* < 0.01, ** *p* < 0.001, ns: not significant.

Previous studies demonstrated that DRAK1 is localized in the nucleus and induces apoptosis when overexpressed [19]. Our results show that DRAK1 is transcriptionally induced by RNase L activation and IFN signaling. We next investigated if overexpression of DRAK1 could induce apoptosis in the absence of RNase L activation. PC3 cells transfected with Green Fluorescent Protein (GFP)-DRAK1 or GFP alone as control were visualized microscopically at various time points. At 16 h post transfection, GFP-DRAK1 localized to the nucleus compared to diffuse cytosolic staining of GFP expressing cells and 24 h later GFP-DRAK1 expressing cells displayed morphological changes like membrane blebbing characteristic of apoptotic cells (Figure 2A). To further confirm that DRAK1 overexpressing cells show increased apoptosis, cell viability was determined in PC3 cells at various time points after DRAK1 transfection. Cell viability decreased over time and only 23% cells remained viable 36 h post transfection (Figure 2B). Expression of DRAK1 resulted in increase in apoptotic cells with sub-G1 DNA content as determined by analyzing DNA content with fluorescence-activated cell sorting (FACS) and trypan blue exclusion (Figure 2C,D). To determine the physiological levels of DRAK1 that induces apoptosis, PC3 cells were transfected with increasing amounts of DRAK1 plasmid and mRNA levels were compared to 2-5A transfected cells (Supplementary Figure S1A). Apoptosis induced was determined by trypan blue exclusion assay and compared to levels of DRAK1 (Supplementary Figure S1B). To further demonstrate that DRAK1 is involved in RNase L-induced apoptosis, DRAK1 levels were reduced in PC3 cells transfected with DRAK1 short interfering RNA (siRNA) and compared to scrambled control siRNA. siRNA targeting DRAK1 reduced protein levels as no induction was observed following Poly I:C transfection (Figure 2E). To rule out the contribution of DRAK2, siRNA DRAK2 was transfected and cell viability was compared after 2–5A transfection. Cell viability increased 15% when DRAK1 levels were knocked down whereas decreasing DRAK2 levels did not affect cell viability after RNase L activation by 2–5A (Figure 2E–H). Thus, increase in DRAK1 levels is sufficient to induce apoptosis and suggests that DRAK1 induction by RNase L can be an important component of RNase L-induced apoptosis in cells.

### 2.3. Ectopic Expression of DRAK1 Induces Apoptosis by Activating JNK and Cleavage of Caspase-3

**Figure 3:**
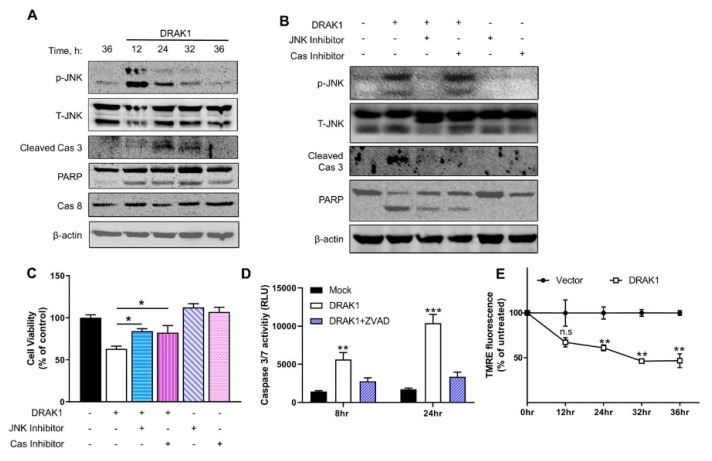
DRAK1 overexpression activates JNK signaling and caspase 3 cleavage to promote apoptosis. (**A**) PC3 cells were transfected with DRAK1 and cell lysates from the indicated time points were immunoblotted with indicated antibodies. PC3 cells were treated with JNK inhibitor (SP600125, 25 μM) or caspase 3 inhibitor (zVAD-FMK, 20 μM) and transfected with DRAK1 for 24 h and (**B**) Cell lysates were probed with indicated antibodies and normalized to β-actin levels, (**C**) Cell viability analyzed by MTT assay. (**D**) PC3 cells were transfected with vector alone or DRAK1 in the presence or absence of caspase 3 inhibitor (zVAD-FMK, 20 μM). Caspase 3/7 activity was measured in cell lysates after 24 h. (**E**) Cells expressing DRAK1 or vector alone were stained with TMRE at indicated times and fluorescence intensity was normalized to control cells. Values shown are mean ± s.d of triplicate assays. * *p* < 0.01, ** *p* < 0.001, *** *p* < 0.0001, n.s: not significant.

To specifically address the role of DRAK1 and signaling pathways involved in apoptosis, further experiments utilized ectopic expression of DRAK1. Next, we investigated the signaling pathways that mediate DRAK1-induced apoptosis. Previous studies show that JNK is activated in RNase L-induced apoptosis accompanied by cleavage of PARP and caspase-3 [17,27]. Thus, we examined if ectopic expression of DRAK1 could activate JNK signaling in PC3 cells transfected with DRAK1. Phosphorylation of JNK that is required for its activation was evident 12h after transfection and significant phosphorylation was detected until 36 h. Increase in JNK activity correlated with cleavage of key apoptotic proteins, PARP and caspase 3, which is a hallmark of intrinsic mitochondrial pathway of apoptosis. No cleavage of caspase 8, an extrinsic pathway substrate was observed under similar conditions (Figure 3A). To determine if DRAK1 is required for JNK phosphorylation, we analyzed phospho-JNK and cleavage of caspase 3 in DRAK1 knockdown cells treated with 2-5A. We observed decrease in both phosphorylation of JNK and caspase 3 cleavage which correlated with increase in cell viability and decrease in apoptosis (Figure 2E–H).

To determine whether JNK activity is required for apoptosis, we examined the effect of SP600125, a JNK inhibitor, in PC3 cells over-expressing DRAK1. Treatment of PC3 cells with SP600125 resulted in inhibition of JNK phosphorylation in cells expressing DRAK1, which correlated with absence of caspase 3 cleavage and decrease in PARP cleavage (Figure 3B, lane 3). Treatment with carbobenzoxy-valyl-alanyl-aspartyl-[O- methyl]- fluoromethylketone (ZVAD-FMK), a pan caspase inhibitor, blocked caspase 3 cleavage, but not JNK phosphorylation indicating that JNK activation by DRAK1 is upstream of caspase 3 function (Figure 3B, lane 4). We further quantitated cell viability in PC3 cells overexpressing DRAK1 following treatment with JNK inhibitor or caspase 3 inhibitor by MTT assay. Consistent with our results, we observed increase in cell viability in DRAK1 expressing cells when we inhibited activity of JNK and cleavage of caspase 3 (Figure 3C). To further demonstrate that caspase 3 cleavage is required for apoptosis in DRAK1 expressing cells, we measured caspase 3 enzyme activity using a luminescent caspase 3 substrate. Cell lysates from DRAK1 over-expressing cells treated with ZVAD-FMK inhibitor showed 2 to 3-fold reduced caspase 3 enzyme activity (Figure 3D). Mitochondrial pathway of apoptosis involves cleavage of caspase 3 along with release of cytochrome C from the mitochondria and loss of transmembrane potential [28] which can be measured by uptake of fluorescent dye, tetramethylrhodamine ethyl ester (TMRE). DRAK1 expressing PC3 cells showed progressive loss of mitochondrial membrane potential (less than 50% compared to control cells) suggesting that DRAK1 induces apoptosis by mitochondrial damage (Figure 3E). Taken together, these results suggest that ectopic expression of DRAK1 activates JNK that is required for cleavage of key apoptotic inducers, caspase 3 and PARP accompanied by loss of mitochondrial transmembrane potential resulting in apoptosis.

### 2.4. DRAK1 Kinase Activity and Nuclear Localization Are Required for Inducing Apoptosis

**Figure 4:**
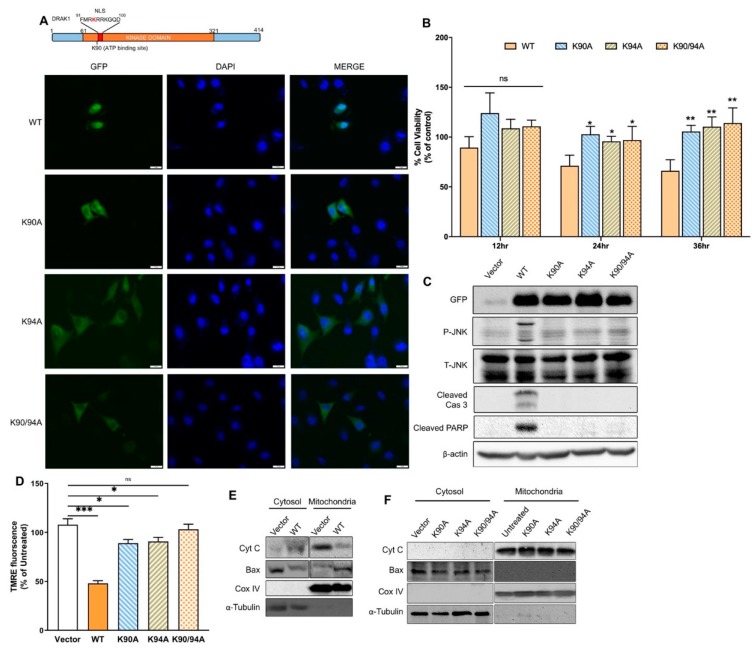
Involvement of DRAK1 kinase activity and nuclear localization in inducing apoptosis. (**A**) Schematic representation of DRAK1 kinase ATP binding site and nuclear localization signal (NLS). PC3 cells expressing GFP-WT or DRAK1 mutants analyzed by immunofluorescence microscopy (scale bar, 20 µm, representative images are shown), (**B**) Cell viability analyzed by MTT assay at indicated times following transfection, (**C**) Cell lysates probed with indicated antibodies and normalized to β-actin levels, (**D**) Cells were stained with TMRE and fluorescence intensity was normalized to control cells. (**E**,**F**) PC3 cells were transfected with GFP-WT or DRAK1 mutants and 24 h later mitochondrial and cytosolic fractions were isolated and analyzed as indicated. Values shown are mean ± s.d of triplicate assays. * *p* < 0.01, ** *p* < 0.001, *** *p* < 0.0001, n.s: not significant.

DRAK1 was localized in the nucleus and amino acid sequence analysis identified a highly conserved nuclear localization sequence (NLS) in the N-terminal kinase domain that overlapped with the conserved lysine residue in the ATP-binding site of DRAK1 (Figure 4A). To evaluate the role of kinase activity and nuclear localization of DRAK1 in inducing apoptosis, we constructed three mutants in which (a) a critical lysine in the ATP binding site required for kinase activity was mutated to alanine (K90A), (b) a conserved lysine in the putative NLS sequence was mutated to alanine (K94A) or (c) both lysine residues were replaced with alanine (K90A/K94A). The GFP-tagged mutant proteins were expressed in PC3 cells to determine the subcellular localization and compared to WT DRAK1. The nuclei were simultaneously visualized by staining with 4′,6-diamidino-2-phenylindole (DAPI). As shown in Figure 4A, the fluorescent staining of DRAK1 WT overlapped with the nuclear staining, while the kinase-dead (K90A), NLS mutant (K94A), or both kinase and NLS mutant localized to the cytoplasm (Figure 4A). These results suggest that kinase activity of DRAK1 is required for its nuclear localization and NLS mutant which has kinase activity does not localize to the nucleus.

To investigate the correlation between kinase activity and nuclear localization with initiation of apoptosis, we expressed DRAK1 or the mutant constructs in PC3 cells and quantitated cell viability in time course experiments. Expression of WT DRAK1 reduced cell viability to 57% when compared to control cells with vector alone. In contrast, viability of cells expressing kinase-dead, NLS mutant and combining both kinase and NLS mutant restored viability to same level as control cells (Figure 4B). Furthermore, DRAK1-induced JNK activity and cleavage of caspase 3 and PARP was reduced in cells expressing the DRAK1 mutants (Figure 4C) which correlated with reduced mitochondrial damage and transmembrane potential (Figure 4D).

We have shown that RNase L activates JNK and promotes switch from autophagy to apoptosis involving Bax translocation to the mitochondria and release of cytochrome C to the cytosol [16]. One major mechanism of activation and cleavage of caspase 3 involves release of cytochrome C from the mitochondria. Since ectopic DRAK1 expression activates JNK that is required for apoptosis, we tested Bax translocation to the mitochondria and release of cytochrome C. Following subcellular fractionation, Bax translocation to the mitochondria was detected on immunoblots only in WT DRAK1 expressing cells compared to control cells and this was accompanied by release of mitochondrial cytochrome C to the cytoplasm. Whereas in DRAK1 mutants that lacked apoptosis-inducing function, Bax remained cytosolic along with retention of cytochrome C in the mitochondria (Figure 4E,F). Taken together, our results indicate that DRAK1 activates JNK signaling and induces apoptosis involving translocation of Bax to the mitochondria suggesting that DRAK1 acts upstream of mitochondrial-based pro-apoptotic proteins. Furthermore, both kinase activity and its nuclear localization are required for inducing cell death as mutants lacking both kinase and nuclear localization lacked apoptosis-inducing function.

## 3. Discussion

Antiviral activity of RNase L involves apoptotic clearance of virus-infected cells and cleaving diverse RNA substrates to produce IFN, activate inflammasome or autophagy [13,14,15,16]. Our studies have identified DRAK1, a DAP kinase family member, as a component in the apoptotic pathway induced by activation of RNase L. RNase L induces expression of DRAK1 and requires IFN signaling pathway suggesting that DRAK1 is an interferon stimulated gene. The presence of ISRE sequences in DRAK1 promoter further support this conclusion. DRAK1 is localized to the nucleus and ectopically expressing DRAK1 is sufficient to trigger apoptosis in the absence of RNase L activation. Knockdown of DRAK1 levels, but not DRAK2, with siRNAs significantly reduced apoptosis induced by RNase L. Furthermore, ectopic DRAK1 expression triggered apoptosis by activating JNK and translocation of Bax to the mitochondria accompanied by cleavage of caspase 3 and PARP. Finally, we show that kinase activity and nuclear localization is required for DRAK1 induced apoptosis. Together, these data indicate that DRAK1 functions as an effector of RNase L induced apoptosis.

Given the complexity of apoptosis pathways activated by RNase L, we overexpressed DRAK1 to study the direct effects in promoting apoptosis in the absence of RNase L activation. We show that ectopic expression of DRAK1 induces cell death involving the activity of JNK and cleavage of caspase 3 and PARP. Inhibiting the activity of JNK using small molecule inhibitor in cells expressing DRAK1 abrogates caspase 3 cleavage and apoptosis. JNK1/2 is required for apoptosis in response to diverse cytotoxic and genotoxic stress stimuli [29,30,31,32,33]. JNK phosphorylates Bax and activated Bax translocates to the mitochondria to promote mitochondrial outer membrane permeabilization resulting in release of cytochrome C that in turn triggers caspase 9 cascade leading to cleavage of caspase 3 and consequent cellular apoptosis [34,35]. While we were unable to detect phosphorylation of Bax, here we show that ectopic DRAK1 expression induced translocation of Bax from cytoplasm to mitochondria and release of mitochondrial cytochrome C accompanied by loss of mitochondrial membrane potential (Figure 3,4). Recently, another study showed interaction of DRAK1 with ANT2, a mitochondrial protein and translocation of DRAK1 to the cytosol in response to PKC activation, however, the impact on apoptosis was not explored [24]. In the IFN signaling pathway, Bax has been shown to bind Interferon Regulatory Factor 3 (IRF3) and translocate to mitochondria and induce apoptosis in virally infected cells [36]. Our previous studies show a similar translocation of Bax in response to cleavage of Beclin-1 and insertion of cleaved Beclin-1 in the mitochondria to induce apoptosis [16]. Future studies will test if JNK is phosphorylated directly by DRAK1 and the consequences on cellular localization of both JNK and DRAK1. IFN signaling and apoptosis are complex pathways and apoptosis can be induced by several components of the IFN pathway, including RNase L. It is likely many other factors may contribute to cell death and further studies are required to investigate their interactions.

The kinase domain of all members of the DAPK family share significant homology and the regulatory regions are structurally diverse indicating death-inducing roles in response to diverse stimuli [37]. DAPK and Zipper-Interacting Protein Kinase (ZIPK) participate in apoptosis in response to IFN-γ, Tumor Necrosis Factor-alpha (TNF-α), Fas ligand and TGF-β, while DRAK2 mediates apoptosis in response to UV treatment and interleukin-2 [38,39]. In this study, we show that DRAK1 is induced in response to type I IFN and dsRNA. DAPK localizes to the cytoplasm, whereas ZIPK, DRAK1 and DRAK2 localize to the nucleus. Both DRAK1 and DRAK2 share 67% identity in the kinase domain and 24% identity in the regulatory domains suggesting differential regulation. Consistent with other studies, DRAK1 kinase-dead mutant (K90A) localized to the cytoplasm in PC3 cells. An earlier study reported that kinase activity of both DRAK1 and DRAK2 is not required for nuclear localization but is required for inducing apoptosis [19]. However, a recent study showed that DRAK2 nuclear localization and increase in kinase activity is required for cell death [38,40]. Our study also identified a short stretch of basic residues RKRRK (aa residues 93–97) in the DRAK1 kinase domain that functions as a nuclear localization signal as K94A mutation resulted in cytoplasmic retention of DRAK1. A corresponding region in DRAK2 (KKRRR, aa 65–69) functions as a NLS for DRAK2 in Jurkat cells [41]. Another putative NLS was identified SKRFK (aa residues 395–399) in the C-terminus of DRAK1 that overlapped with PKC phosphorylation site at S395 [24]. Similarly, a second NLS in the C-terminal of DRAK2 was identified which was regulated by phosphorylation of Ser350 in the C-terminal region [40]. Kinase activity and nuclear localization of DRAK1 is required for inducing apoptosis as DRAK1 kinase-dead, NLS mutant or kinase-dead and NLS double mutant showed reduced apoptosis when ectopically expressed in PC3 cells. The kinase-dead mutant had similar ability to rescue cell viability as NLS mutant or double mutant suggesting that both nuclear localization and kinase activity of DRAK1 is perhaps integral to its apoptosis-inducing activity. While we have observed variability in levels of apoptosis in comparing DRAK1 WT and mutants, we consistently observed lack of JNK phosphorylation in all mutants. It is unclear if DRAK1 phosphorylates JNK in the nucleus and if there are additional substrates in the nucleus that are phosphorylated and needed for signaling. In that regard, we have not explored if DRAK1 is autophosphorylated and if trans-phosphorylation of JNK is compromised by the mutants we have studied. It is also likely that other substrates may be phosphorylated in the nucleus that promotes apoptosis. In Human osteosarcoma (U2OS) cells, DRAK1 bound to p53 in the nucleus and cisplatin-induced phosphorylation of p53 was reduced in DRAK1 knockdown cells [24]. Further investigations will reveal if DRAK1 directly or indirectly regulates p53 and its apoptotic activities. Many tumor cells, including PC3, have mutant p53 protein so the effects we observed are p53-independent. Future studies will be aimed at resolving the distinct roles of DRAK1 as a kinase and as a nuclear localized apoptosis-inducing protein.

In addition to IFN and dsRNA, DRAK1 is induced in a p53-dependent manner in response to DNA damage agents in testicular, ovarian and pancreatic cell lines [20]. Cells with increased expression of DRAK1 were more susceptible to cell death with chemotherapeutic drugs [21]. In testicular cancer cells, DRAK1 is induced in response to cisplatin and induces cell death by modulating Reactive Oxygen Species (ROS) activity. However, in Head and neck squamous cell carcinoma (HNSCC) cell lines, where DRAK1 is overexpressed, DRAK1 in the cytoplasm binds to Smad3 and sequesters it in the cytoplasm, thereby preventing Smad3/Smad4 complex formation and inhibiting TGF-β1 tumor suppression [23]. These results suggest that DRAK1, predominantly, has anti-tumor functions and agents like IFN and dsRNA that can induce DRAK1 expression can potentially be combined for effective cancer therapy.

## 4. Materials and Methods

### 4.1. Chemicals, Reagents and Antibodies

Chemicals, unless indicated otherwise, were from Sigma Aldrich (St. Louis, MO, USA). Antibodies to phospho-stress-activated protein kinase (SAPK)/JNK (Thr^183^/Tyr^185^), total JNK, Caspase 3, caspase 8, PARP, CoxIV, Cytochrome C were from Cell Signaling, Inc. (Danvers, MA, USA). Antibodies to GFP were from Thermo Scientific (Waltham, MA, USA), β-actin and DRAK1 were from Sigma (St. Louis, MO, USA), DRAK2 and Bax were from Santa Cruz Biotechnology (Santa Cruz, Dallas, TX, USA). Anti-mouse IgG and anti-rabbit IgG HRP linked secondary antibodies were from Cell Signaling, Inc. and ECL reagents were from GE Healthcare (Piscataway, NJ, USA) and Boston Bio products (Ashland, MA, USA). The JNK inhibitor SP600125 (Millipore-Sigma, Burlington, MA, USA), propidium iodide, ribonuclease A (Sigma-Aldrich), and zVAD-FMK (Santa Cruz Biotechnology) were prepared as suggested by the manufacturers and used at indicated concentrations. Preparation of 2–5A using ATP and recombinant 2–5A synthetase (a generous gift from Rune Hartmann, University of Aarhus, Aarhus, Denmark) has been described previously [15]. Poly I:C was purchased from Calbiochem (San Diego, CA, USA).

### 4.2. Cell Culture and Transfections

Prostate cancer cells PC3 and DU145 were grown in RPMI 1640 supplemented with streptomycin (100 μg/mL), penicillin (100 units/mL), 2 mmol/L glutamine, and 10% fetal bovine serum (Sigma-Aldrich). The human fibrosarcoma cell line, HT1080 (a gift from Ganes Sen, Cleveland Clinic, Cleveland, OH, USA), U3A (STAT1-defective, derived from parental 2fTGH cells, which were derived from HT1080 cells (a gift from George Stark, Cleveland Clinic, Cleveland, OH, USA) and 293T, were cultured in Dulbecco’s modified minimal essential medium with 10% fetal bovine serum (Sigma-Aldrich), 100 μg/mL penicillin/streptomycin, 2 mM L-glutamine, and non-essential amino acids. Cells were maintained in 95% air, 5% CO_2_ at 37°C. Transfection of 2–5A (10 µM) was performed using Lipofectamine 2000 (Invitrogen) according to the manufacturer’s protocol. Briefly, cells were plated one day before transfection, so that the cells are 80–90% confluent at the time of transfection. 2–5A was diluted into serum-free media and then mixed with Lipofectamine 2000 for 20 min before being added to cells in growth media. Poly I:C (2 μg/mL), plasmids were transfected into cells using Polyjet reagent (SignaGen Laboratories, Gaithersburg, MD, USA). For experiments involving inhibitors, cells were transfected with DRAK1-GFP plasmid in 10 cm culture dishes, then six hours later the inhibitors were added. Transfection of siRNA in PC3 cells were performed using Lipofectamine 2000 reagent (Invitrogen, Carlsbad, CA, USA), according to the manufacturer’s protocols. DRAK1 and DRAK2 targeting siRNA and control siRNA were obtained from Santa Cruz Biotechnology. Knock-down of endogenous DRAK1 or DRAK2 was determined by western blotting.

Construction of RNase L gene knockout cells: The HT1080 RNase L KO cells were generated by using the CRISPR Cas9 system (Available online: https://portals.broadinstitute.org/gpp/public/analysis-tools/sgrna-design). The sgRNA sequences were: RNL F CACCGTTATCCTCGCAGCG ATTGCG and RNL R AAACCGCAATCGCTGCGAGGATAAC targeting exon 1. The guide RNA sequences were synthesized as DNA oligonucleotides and annealed, phosphorylated and ligated into the vector pSpCas9(BB)-2A-Puro (PX459) V2.0 (a gift from Feng Zhang (Addgene plasmid # 62988) that was prepared by digestion with BsmBI [42]). The resulting plasmids were used to transfect HT1080 cells and selected in puromycin. Clones were obtained by limiting dilution and screened for knockout of RNase L expression by immunoblotting. Knockout cell lines were expanded and tested for loss of RNase L enzyme activity following 2-5A transfection and analysis of RNA cleavage using total RNA as previously described [15].

### 4.3. Plasmids

The cDNA encoding the DRAK1-GFP was obtained from the DNASU repository (Arizona State University, Tempe, AZ, USA). Mutant DRAK1 plasmids were synthesized by Biobasic (Toronto, ON, Canada) in pUC57 vector and inserted as Xma I and Kpn I fragments into the pLPS-3′EGFP vector backbone containing the same restriction sites and confirmed by DNA sequencing. Expression of the constructs was confirmed by immunoblot analysis. DNA fragment corresponding to −2718 was synthesized by Genewiz (South Plainfield, NJ, USA) and cloned upstream of luciferase reporter in pGL4-26 vector as Kpn I-Sac I fragment (Promega, Madison, WI, USA). Promoter truncation constructs −2496 (Hind III-Sac I), −1336 (Nhe I-Sac I) and −671 (Bgl II-Sac I) were cloned in pGL4-26 vector and all constructs were confirmed by DNA sequencing.

### 4.4. Cell Viability and Caspase 3/7Assays

Cell viability was determined by MTT assay using the colorimetric CellTiter 96 Aqueous Cell Proliferation Assay (Promega). Briefly, cells (1 × 10^4^ cells per well) were seeded in a 96-well culture plate and transfected with DRAK1-GFP, DRAK1-GFP mutants, Poly I:C (2 µg/mL) or small RNA and control RNA for indicated times. At indicated times 20 µL of CellTiter 96 Aqueous reagent (40% (*v*/*v*) dilution in PBS) was added to each well. Plates were incubated at 37 °C up to 3 h, and absorbance was measured at 490 nm with a 96-well plate reader (model Spectra Max 340; Molecular Devices, Menlo Park, CA, USA). Viability was normalized against mock treated cells. PC3 cells in 10 cm dishes were transfected with GFP vector or DRAK1-GFP and at indicated times cells were harvested, washed twice with PBS, and fixed with ice-cold 70% ethanol at −20 °C for 2 h and intracellular DNA was labeled with propidium iodide as described previously [16]. The percentage of apoptotic cells, sub G1, was determined using FACSCalibur flow cytometer equipped with CellQuest software (Becton-Dickinson, San Jose, CA, USA). For the trypan blue exclusion experiments, cells treated as above were stained in 0.4% trypan blue solution (Life Technologies, Carlsbad, CA, USA) and then counted using a hemacytometer under inverted microscope (Leica Microsystems, Wetzlar, Germany) and normalized to mock treated cells. PC3 cells (1 × 10^4^ cells per well) were grown in black-walled 96-well plates with transparent bottom and transfected with DRAK1 plasmid (50 ng) and at indicated times caspase3/7 activity in lysates was measured using ApoONE homogenous caspase-3 and -7 assay kit (Promega) as described previously. Indicated samples were pretreated with 20 µM of zVAD-FMK for 1h before transfection to inhibit caspase 3/7 activity. Experiments were performed in triplicate, and the results are representative of three independent experiments and shown as ± SD.

### 4.5. Cell Fractionation and Immunoblotting

PC3 cells were harvested following transfection as indicated and cytosolic and mitochondria fractions were prepared as described previously [16]. Proteins of the fractions were separated by SDS-Page and analyzed by immunoblotting. For immunoblot analysis, cells were washed with ice cold PBS and lysed in buffer containing 0.5% NP-40, 90 mM KCl, 5 mM Mg acetate, 20 mM Tris, pH 7.5, 5 mM β mercaptoethanol, 0.1 M PMSF, 0.2mM sodium orthovanadate, 50 mM NaF, 10 mM glycerophosphate, protease inhibitor (Roche Diagnostics, Indianapolis, IN, USA) and analyzed by SDS-Page and immunoblotting using indicated primary antibodies. Membranes were washed and incubated with goat anti-mouse or goat anti-rabbit antibody tagged with horseradish peroxidase (Cell Signaling) and proteins in the blots were detected by enhanced chemiluminescence (GE Healthcare, Chicago, IL, USA).

### 4.6. RNA Isolation and Quantitative Real Time PCR

RNA was isolated using Trizol reagent (Invitrogen, Thermo Fisher Scientific, Waltham, MA, USA) as per the manufacturer’s instructions and used for cDNA synthesis using random decamers and a RETROscript cDNA synthesis kit (Life Technologies; Thermo Fisher Scientific). Expression of DRAK1 and DRAK2 mRNA was determined by quantitative reverse transcription polymerase chain reaction (qRT-PCR) using SYBR Green PCR Master Mix (Bio-Rad Laboratories Inc., Hercules, CA, USA) using the gene-specific primers and normalized to *GAPDH* expression. Primer sequences used are:

DRAK1-F 5′ TGCTGTGTGAACCTGTCAAAGCAC 3′
DRAK1-R 5′ACCTGGTTGTCTGAAGTGCCTGAT 3′
DRAK2-F 5′AAAAATAGGGCATGCGTGTGA 3′
DRAK2- R 5′ CATAGTTCAGGATTTCTGGAGCTAAA 3′
GAPDH F 5′ GCAAATTCCATGGCACCGT 3′
GAPDH R 5′ TCGCCCCACTTGATTTTGG 3′

### 4.7. Luciferase Reporter Gene Assays

PC3 cells were transfected with promoter reporter luciferase constructs (1.0 µg) and Renilla luciferase plasmid (0.1 µg) to normalize transfection efficiency in the absence or presence of 2–5A (10 µM), Poly I:C (2 μg/mL) or type I interferon (1000 units) for 16 h. Luciferase activities were quantified with Dual luciferase activity kit (Promega) and relative luciferase values normalized to control. Experiments were performed in triplicate, and the results are representative of three independent experiments and shown as ± SD.

### 4.8. Tetramethylrhodamine Ethyl Ester (TMRE) Mitochondrial Membrane Potential Assay

2 × 10^4^ cells per well of PC3 cells were plated in a 96-well black plate with clear bottom and transfected with vector, GFP-DRAK1 (WT) or DRAK1 mutant plasmids. After indicated time points, the cells were washed with PBS and TMRE reagent (10 nM in serum-free medium) was added and incubated at 37 °C for thirty minutes and the fluorescence was measured on a plate reader (model Spectra Max 340; Molecular Devices) at 549 nm excitation and 575 nm emission.

### 4.9. Immunofluorescence Microscopy

PC3 cells were transfected with control EGFP plasmid, GFP-DRAK1 or GFP-DRAK1 mutants using Lipofectamine 2000 reagent 24–48 h prior to visualizing cells under fluorescence microscope for characteristics of cell death. Cells were cultured on coverslips prior to transfection and fixed with 4% paraformaldehyde in PBS for 10 min at room temperature and permeabilized with 0.2% Triton X-100 in PBS for 10 min and mounted in Vectashield with DAPI to stain the nucleus (Vector Labs, Burlingame, CA, USA). Fluorescence and confocal microscopy assessments were performed with Leica CS SP5 multi-photon laser scanning confocal microscope (Leica Microsystems).

### 4.10. Statistical Analysis

All values are presented as mean ± SD and are representative of at least three independent experiments. Student’s *t*-tests were used for determining statistical significance between groups and *p* ≤ 0.05 was considered significant.

## 5. Conclusions

In this study, we show that RNase L induces expression of a serine/threonine protein kinase, DRAK1, involving IFN signaling pathway. DRAK1 localizes to the nucleus and overexpression of DRAK1 in the absence of RNase L activation induces apoptosis by activating JNK and promoting Bax translocation to the mitochondria and cleavage of caspase 3 and PARP. Both kinase activity and nuclear localization are required for inducing apoptosis. Our studies identify DRAK1 as a mediator of RNase L-induced apoptosis.

## Supplementary Materials

Supplementary data can be found at https://www.mdpi.com/1422-0067/20/14/3535/s1.
