# Supplementary material for: RNase L Induces Expression of A Novel Serine/Threonine Protein Kinase, DRAK1, to Promote Apoptosis
Source: Int J Mol Sci. 2019 Jul 19;20(14):3535. doi: 10.3390/ijms20143535 (PMC6679093; doi:10.3390/ijms20143535)
Supplement: Supplementary file 1 [file ijms-20-03535-s001.pdf]

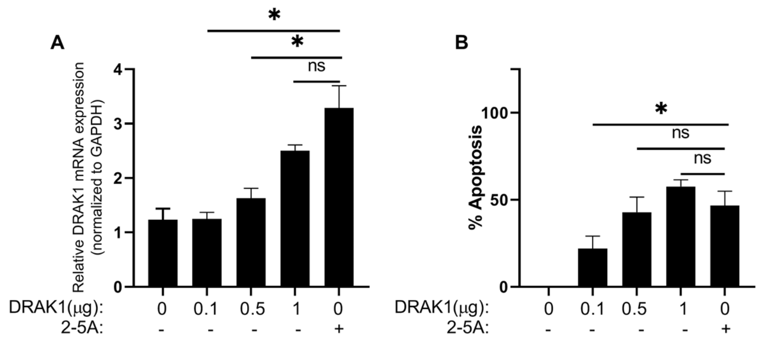

Supplementary Figure 1. PC3 cells were transfected with increasing amounts of DRAK1 plasmid or 10 μM of 2–5A. (**A**) After 24 h, the levels of DRAK1 mRNA were measured by RT-PCR using DRAK1-specific primers and normalized to GAPDH mRNA levels, or (**B**) Apoptosis % was determined by trypan blue exclusion. Data shown are mean values ± SD from experiment performed in triplicate. Student's *t* test was used to determine *p* values. *$p < 0.01$, not significant (ns).
